# Infectious Causes of Cholesteatoma and Treatment of Infected Ossicles prior to Reimplantation by Hydrostatic High-Pressure Inactivation

**DOI:** 10.1155/2015/761259

**Published:** 2015-02-01

**Authors:** Wycliffe Omurwa Masanta, Rebecca Hinz, Andreas Erich Zautner

**Affiliations:** ^1^Institut für Medizinische Mikrobiologie, Universitätsmedizin Göttingen, 37075 Göttingen, Germany; ^2^Fachbereich Tropenmedizin am Bernhard-Nocht-Institut, Bundeswehrkrankenhaus Hamburg, 20359 Hamburg, Germany

## Abstract

Chronic inflammation, which is caused by recurrent infections, is one of the factors contributing to the pathogenesis of cholesteatoma. If reimplantation of autologous ossicles after a surgical intervention is intended, inactivation of planktonic bacteria and biofilms is desirable. High hydrostatic pressure treatment is a procedure, which has been used to inactivate cholesteatoma cells on ossicles. Here we discuss the potential inactivating effect of high hydrostatic pressure on microbial pathogens including biofilms. Recent experimental data suggest an incomplete inactivation at a pressure level, which is tolerable for the bone substance of ossicles and results at least in a considerable reduction of pathogen load. Further studies are necessary to access how far this quantitative reduction of pathogens is sufficient to prevent ongoing chronic infections, for example, due to forming of biofilms.

## 1. Introduction

Cholesteatoma is a noncancerous condition that is characterized by abnormal growth of squamous epithelial cells in the middle ear and mastoid destroying the ossicles resulting in loss of hearing. This condition affects children more aggressively than adults. There are two types of cholesteatoma, namely, congenital and acquired cholesteatoma. General symptoms include release of smelly fluid from infected ear, loss of hearing, and pain on the infected ear [1]. Treatment is a combination of surgery and the administration of antimicrobials [2, 3]. However, sometimes recovery is complicated by postsurgery infections [4].

The etiology of cholesteatoma is not yet completely understood, but various studies have revealed that a number of factors cooperate in a synergistic way to cause the forming of this nonneoplastic keratinizing lesion, which is characterized by enhanced proliferation of epithelial cells with aberrant morphologic characteristics [1, 5–7]. These factors include persistent microbial infection resulting in chronic inflammation, consecutive invasion by cells of the immune system, Eustachian tube dysfunction, aggregation of cellular debris, and increased viscosity of middle ear effusions, in-growth of blood vessels, auditory ossicle resorption, and epithelial hyperplasia [7, 8].

During surgical treatment of cholesteatoma, affected parts of the ossicular chain must be removed. Because of best recovery of hearing autologous retransplantation of ossicles is still the therapy of choice [9, 10]. Therefore, devitalization of cholesteatoma-affected parts of the ossicular chain and their reimplantation would be beneficial. The hydrostatic high-pressure technology (HHD) is a promising method that can remove the cellular components during ongoing surgery. In the food industry, high pressure is already used as a substitute for pasteurization. Microorganisms are inactivated by high-pressure, but the required pressure level depends on the respective germ [11, 12]. The mechanism of action of hydrostatic high-pressure is essentially based on the changes taking place at the phase boundary of water to other molecules, in particular, the epithelial or bacterial cell surface. Under increasing pressure water molecules penetrate into the cavities of complex macromolecules and blow up the quaternary and tertiary structure of complex macromolecules. Covalent bonds are unaffected since the primary structure is not changed by high-hydrostatic pressure [13, 14].

This paper will give a detailed overview on persistent microbial infections associated with cholesteatoma and their elimination by hydrostatic high pressure (HHP) during surgery to minimize the possibility of postsurgery infection.

## 2. Persistence of Microbial Pathogens in spite of Surgical Therapy of Cholesteatoma

Colonization by bacteria leads to biofilm formation on the ossicles of the middle ear. These biofilms lead to impaired clearance and consecutive chronic middle ear infection, which triggers chronic inflammation. Mediators expressed during inflammation like IL-1, PAF, and TNF-alpha induce mucin hypersecretion, hyperproliferation of epithelial cells and keratinocytes and bone resorption by activation of collagenases and osteoclasts [7].

Quite a number of Gram-positive, Gram-negative, and fungal pathogens have been isolated from cholesteatoma tissues (please see Table 1) [15–23]. Biofilm formation is believed to play an important role in persistence of these pathogens in the middle ear hence maintaining chronic inflammation eventually leading to the establishment of cholesteatoma [17, 18]. A recent* in vitro *study revealed that biofilm was responsible for the persistence of more than 50% of isolates of microbial pathogens in ossicles that were obtained from a cholesteatoma tissue [23]. The great variety of suspected relevant pathogens suggests that the general inflammatory stimulus due to bacterial infections seems to be more important than the causing bacterial species itself. Further, as the bacterial flora of the upper respiratory tract physiologically colonizes the middle ear cavity, it is difficult to discriminate relevant pathogens from harmless colonizers.

As we stated in the introduction, treatment of cholesteatoma is usually based on surgery [2, 3, 24–28], including the surgical removal of chronically inflamed ossicles. Relapses are frequent with recurrence rates <10% already being considered as therapeutic success [4, 29–31]. Incomplete removal of pathogens or their biofilms, in particular, is a proven risk factor for recurrence [31].

For the treatment of middle ear cholesteatoma, reimplantation of autologous ossicles is frequently applied [9, 10, 32] because optimal recovery of hearing is hardly achieved by allogeneic implants despite good biocompatibility and stability. The implantation of fixated homologue ossicles from an “ossicle bank” bears the potential risk of slow-virus or prion transmission and is, therefore, critically discussed [33].

Cholesteatoma cells on autologous ossicles should be thoroughly inactivated prior to any reimplantation approaches. The surgeon should abstain from immediate reimplantation if a readily removable coat of cholesteatoma cells or an infiltration by cholesteatoma-matrix into the bone is observed during surgery [34, 35].

Next to cholesteatoma cells and difficult to identify, microbial biofilms that have contributed to chronic inflammation finally leading to cholesteatoma formation may persist on explanted ossicles as well [23].

## 3. Hydrostatic High-Pressure (HHP) to Devitalize Human Cells on Explanted Ossicles

Hydrostatic high-pressure technology (HHP), which can effectively disturb or even completely destroy eukaryotic cell membranes, elements of the cytoskeleton, and enzyme systems [36–38], allows for the inactivation of cholesteatoma cells on the ossicles. Previous studies have demonstrated successful devitalizing of bone tissue with intact bone matrix by HHP [39, 40]. Bone and tendon materials [41, 42] are resistant to pressure up to 600 MPa without measurable alteration of their biomechanical properties. HHP efficiently destroys vital human cells without affecting rigid structures of bone tissue [13, 14, 43]. For explanted ossicles, a thorough eradication of vital cholesteatoma cells by pressure of 400 MPa has previously been demonstrated. The cellular damage was mainly caused by extensive membrane disruption [44]. Accordingly, HHP allows the surgeon to destroy any harmful vital human cellular components of ossicle interponates within the surgical procedure.

Infectious complications and persistence of inflammation-inducing biofilms are, however, other risks of reimplantation of ossicles during cholesteatoma surgery. Inactivation of respective microorganisms is, therefore, another point of concern.

## 4. Hydrostatic High-Pressure (HHP) to Devitalize Microbial Pathogens

Several studies analyzed the inactivation of microorganisms by HHP application comprising both bacteria [11, 12, 45] and viruses [46] in food samples. By doing so, the procedure can replace pasteurization which is commonly applied in food industry. The target species defines the required pressure, which is necessary for a thorough inactivation within the sample [11, 12]. In contrast, data on the effects of HHP on colonizing or infecting pathogens in human samples are still rather scarce.

**Figure 1:**
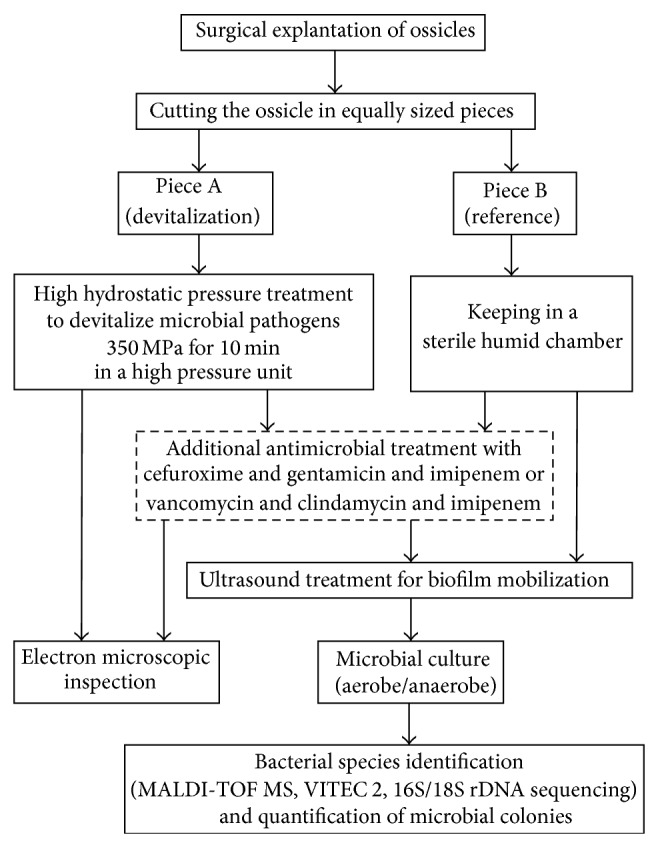
Flow chart of the experimental setup for high hydrostatic pressure treatment on human ossicles. After surgical explanation of human ossicles the bones were cut into two equally sized pieces but in case of additionally antibiotic treatment the bones were cut into six equally sized pieces. Piece(s) A was/were HHP-treated at 350 MPa for 10 min, while Piece(s) B was/were kept in a sterile humid chamber. Optionally, the pieces were treated with either cefuroxime 11.1 mg/mL, gentamicin 44.4 mg/mL, and imipenem 3.7 mg/mL or vancomycin 11.1 mg/mL, clindamycin 0.75 mg/mL, and imipenem 3.7 mg/mL. After treatment, microbial colonization was assessed by electron microscopy and microbial culture. Prior microbial culture bacterial biofilms were mobilized by ultrasound treatment. Microbial species identification was performed using MALDI-TOF MS (Bruker Daltonics, Bremen, Germany), VITEC 2 identification (bioMérieux, Nürtingen, Germany), and 16S/18S rDNA sequencing. For in-depth reading, see [23, 44].

In a recently published study, a moderate inactivating effect of HHP of 350 MPa about 10 minutes, which had been shown to eradicate cholesteatoma cell growth on explanted human ossicles without harming the ossicle itself [44], was shown for colonizing microbes (scheme of experimental setup in Figure 1) [23]. In this study, those HHP conditions allowed for a complete inactivation of bacteria in about half of the tested clinical samples and a thorough eradication of vital cholesteatoma cells. The result was not unexpected as different bacterial species show varying susceptibilities to HHP [12, 47]. Furthermore, nonhomogenously inactivating effects have been observed even within defined species, namely,* Corynebacterium pseudodiphtheriticum*,* Propionibacterium acnes*,* Staphylococcus aureus*,* Staphylococcus hominis*,* Staphylococcus simulans*,* Staphylococcus caprae*, and* Turicella otidis*. For these bacterial species 350 MPa for 10 minutes are close to the their inactivation threshold, therefor semi-quantification of bacterial load after pressure treatment yields equal or even higher amount compared to untreated specimen [23]. Marked differences upon susceptibility against HHP within a species have been described so far [48, 49] with varying numbers of resistant subpopulations within a given strain [50]. As expected due to the protective effects of the thicker cell wall, Gram-positive strains demonstrate a higher HHP resistance than Gram-negative ones [23]. But there are differences within the Gram-negative bacteria as well. While* P. aeruginosa* is readily inactivated by HHP treatment as shown in various prior studies [23, 51], the nonfermenting Gram-negative, rod-shaped* Acinetobacter *spp. resist in a similarly efficient way as staphylococci [23].

## 5. Known Effects of High Hydrostatic Pressure on Microorganisms

Few of the obviously complex mechanisms of bacterial adaption to pressure are analyzed on molecular level, usually for model organisms like lactic acid bacteria. Mesophile bacteria like lactic acid bacteria can be inactivated at 200–600 MPa about 5–60 minutes. If pressure between 200 and 300 MPa is applied, the sigmoid killing curves usually lead to plateaus indicating resistant fractions within the populations [50]. However, adaptive strategies may vary between individual species. Genetic variability was shown to be a factor affecting HHP susceptibility [52]. The pathways of stress response to high pressure are a partial composition of stress response reactions to other stress qualities [53] and show measurable effects on expression or hydratization levels of molecules. High pressure especially alters many macromolecules while small molecules usually remain unchanged. Especially the cellular membrane is altered by the thermodynamic effects of high pressure including decreases of fluidity and integrity as well as changes of secondary and tertiary structure of membrane proteins. Thus deep-sea bacteria like* Photobacterium profundum* require alternative flagellar and porin system for their existence under high-pressure conditions [54]. But also macromolecular associations, that are necessary for cellular division, dissociate under high-pressure conditions due to changes in hydratization [50]. Translation and transcription are affected as well leading to the production of dysfunctional proteins within the cell [55]. In addition, HHP efficiency could be affected by factors such as microbial growth phase, prior exposure to sublethal stress conditions, as well as environment composition and conditions [23].

## 6. Resistance of Bacterial Pathogens against High Hydrostatic Pressure

Bacteria are able to recover in spite of drastically decreased viability due to HHP under favourable conditions [49, 56–60]. It is further known that prior exposure towards sublethal stresses may dramatically increase resistance against HHP even in stress-sensitive microorganisms like* Campylobacter jejuni* [45, 61–63]. HHP-resistant variants of* Listeria monocytogenes* were described to be 10 to 600,000 times more resistant than the wild-type when exposed to 350 MPa [48]. The population diversity of stress resistant* Listeria monocytogenes* variants suggests a high degree of genetic flexibility [52]. Previously observed lacking inactivation of* P. aeruginosa* and* S. epidermidis* in higher concentrations (about MacFarland 0.5) [23] is not surprising, for pressures about 900 MPa about 5 minutes are required for a 8-9 decadic log units reduction as demonstrated for* Staphylococcus*,* Listeria*, and* Salmonella* [60]. Various conditions affect the inactivating effect of HHP treatment, including medium composition, pH value, temperature, ion concentrations (especially magnesium and calcium), sucrose concentration within the medium, growth phase of the microorganisms, and number of compression cycles [45, 49, 56–62]. The analyses of all these interfering factors will require more and larger studies if adequate models for ossicle tissue are available [23].

## 7. Experience of Other Medical Disciples regarding High Hydrostatic Pressure Application for the Inactivation of Microbes on Biological Material

Allogeneic bone transplantation comprises a risk of infection [64]. Previously described experiments with high hydrostatic pressure application about 600 MPa to human bone samples led to a complete disinfection in no more than 2 of 37 bone samples from patients with chronic osteomyelitis [51]. Even in artificially infected bone specimens complete disinfection was achieved in no more than 66% for* Staphylococcus aureus*, 60% for* P. aeruginosa*, and 0% for* Enterococcus faecium*. Interestingly, blood and adherence to metal implants did not significantly alter the inactivating effect of HHP treatment, so quantitative reductions of vital bacteria about 5 decadic log units were achieved for* S. aureus* and* P. aeruginosa*. Nevertheless, the baroprotective effect on osteoarthritic bone was nonhomogeneous. Microorganisms on individual bone samples showed resistance against treatment resulting in unaltered bacterial growth [51]. In a further study, destruction of cell-wall integrity of Gram-negative strains was observed by electron microscopy, but only 71% of bone biopsies from patients with chronic bone infections were culture-negative after high hydrostatic pressure up to 600 MPa compared with 38% negative samples without any treatment [64].

## 8. Combined Effects of High Hydrostatic Pressure and Antibiotic Drugs on the Inactivation of Bacteria

An increase in the inactivating effect of HHP on bacterial organisms in combination with different antibiotic mixtures could be confirmed for ossicle material [23]. In the respective study, the addition of antibiotics suppressed bacterial growth in culture media as might have been expected. The used antibiotic combinations in combination with HHP led to convincingly better inactivating effects than HHP alone.

Nevertheless, there is most certainly no combination of antibiotic agents that could guarantee antimicrobial effects on each possible bacterial species that could colonize the clinical material. In the mentioned study [23], the combination of vancomycin, clindamycin, and imipenem failed to inactivate a* Leuconostoc mesenteroides* ssp. cremoris isolate due to its intrinsic resistance to vancomycin [65]. However, considering the fact that this isolate grew no earlier than after 7 days in an enrichment broth after HHP treatment combined with antibiotics while there was no growth from the same ossicle after HHP treatment alone (personal communication with the authors of [23]), a secondary contamination cannot completely be excluded as well.

However, such laboratory results are difficult to interpret. Transport time from the pressure device to the microbiological laboratory that would be absent in case of an immediate reimplantation after pressure treatment might have an effect due to a prolonged exposure time to the antibiotic drugs. During transport, antibiotic substances can act much longer than the pressure itself. Further, exposure to subletal pressure is known to alter susceptibility to antibiotics, especially to substances acting at the ribosomal subunits [50].

## 9. Effects of High Hydrostatic Pressure on Biofilms

The efficiency of HHP treatment on bacterial pathogens is known to be affected by biofilm formation [52]. Biofilm growth in per se nonsterile compartments like the middle ear cavity has to be expected. Both ossicles and ossicular prostheses can harbour biofilms formed by typical colonizing bacteria of the middle ear cavity in chronically infected or colonized patients [66–68]. Respective* in vivo* experiments are difficult to design, because* in vivo* ability of biofilm formation is only poorly reproducible* in vitro* on a cover-slide. So* in vitro* growth in biofilms does not per se guarantee biofilm formation* in vivo* as well. In a recent study on biofilm-forming isolates from human ossicles, indeed biofilms were less susceptible to HHP treatment than planktonic bacteria as could have been suspected. At least doubling of pressure settings was necessary to eradicate similar bacterial cell quantities in biofilms as in the planktonic state for both Gram-positive and Gram-negative bacteria [23]. Interestingly, the biofilm formed by the Gram-negative pathogen was more HHP-resistant than the biofilm of the Gram-positive bacterium, thus neglecting the importance of cell wall thickness in the biofilm state.

One might speculate whether an ultrasound treatment of ossicles prior to HHP treatment might strengthen the inactivating pressure effects. To the authors' knowledge, no respective studies are currently available. However, the practicability of a multistep-procedure including an ultrasound pretreatment in the clinical setting during a middle ear operation has to be doubted.

## 10. Conclusions

Biofilms play a major role in the development of cholesteatoma. In addition, biofilms on reimplanted ossicles maintain chronic infectious stimuli, which may finally contribute to recurrence of cholesteatoma [7]. According to current state of science, HHP fails to demonstrate reliable inactivation of colonizing microorganisms on ossicles by pressure conditions that have proved to be sufficient to inactivate cholesteatoma cells [23]. However, a reduction of colony forming units due to moderate HHP about several decadic logarithmic units for both planctonic bacteria and biofilms have been described for colonizers of the upper respiratory tract [23]. It remains unclear whether such a reduction of colony forming units may be sufficient in the per se nonsterile middle ear compartment to prevent severe infections or biofilm forming, other than in situations when sterile work is required like in bone and joint operations [51].

The inactivating effects of HHP may be facilitated by the presence of antimicrobial agents [23]. However, no composition of antibiotic drugs may cover the whole spectrum of potential resistance patterns, so surviving colonies cannot be excluded. Further studies on HHP combined with antibiotic drugs are desirable to identify optimal combinations in the future.

At present, it remains unclear whether ossicle tissue would tolerate relevant increases of pressure to levels that might allow for a more complete eradication of microbial agents. Moderate HHP treatment is suitable to reduce the number of microorganisms that colonize ossicles but fails to ensure a reliable sterilization.

## Figures and Tables

**Table 1:** Bacterial and fungal species isolated from cholesteatoma material according to [9–17].

Subgroup	Species
Gram-positive aerobic cocci	*Kocuria rosea *
	*Leuconostoc mesenteroides* ssp. *cremoris *
	*Micrococcus luteus *
	*Staphylococcus aureus *
	*Staphylococcus auricularis *
	*Staphylococcus capitis *
	*Staphylococcus epidermidis *
	*Staphylococcus hominis *
	*Staphylococcus simulans *
	*Streptococcus mitis *
	*Streptococcus sanguinis *

Gram-positive aerobic rods	*Bacillus licheniformis *
	*Corynebacterium pseudodiphtheriticum *
	*Turicella otitidis *

Gram-positive anaerobic cocci	*Peptostreptococcus* spp.

Gram-positive anaerobic rods	*Clostridium bifermentans *
	*Eubacterium limosum *
	*Fusobacterium* spp.
	*Propionibacterium acnes *
	*Propionibacterium granulosum *

Gram-negative aerobic cocci	*Neisseria sicca *
	*Neisseria subflava *

Gram-negative aerobic rods	*Aeromonas salmonicida *
	*Acinetobacter baumannii *
	*Burkholderia cenocepacia *
	*Brevundimonas diminuta *
	*Histophilus somni *
	*Pseudomonas aeruginosa *
	*Pseudomonas fluorescence *
	*Ralstonia pickettii *
	*Sphingomonas paucimobilis *

Gram-negative anaerobic cocci	*Veillonella parvula *

Gram-negative anaerobic rods	*Bacteroides ureolyticus *
	*Porphyromonas* spp.
	*Prevotella* spp.

Yeasts	*Candida albicans *
